# In Utero Exposure to Aluminium and Other Neurotoxic Elements in Urban Coastal South African Women at Delivery: An Emerging Concern

**DOI:** 10.3390/ijerph17051724

**Published:** 2020-03-06

**Authors:** Halina B. Röllin, Kalavati Channa, Bukola Olutola, Claudina Nogueira, Jon Ø. Odland

**Affiliations:** 1 School of Health Systems and Public Health, Faculty of Health Sciences, University of Pretoria, Pretoria 0002, South Africa; Bukola.Olutola@gmail.com (B.O.); claudina.nogueira@up.ac.za (C.N.); jon.o.odland@ntnu.no (J.Ø.O.); 2 Environment and Health Research Unit, Medical Research Council, Johannesburg 2094, South Africa; 3 Lancet Laboratories, Department of Analytical Chemistry, Johannesburg 2090, South Africa; kalavati.channa@lancet.co.za; 4 Department of Community Medicine and Nursing, Faculty of Health Sciences, Norwegian University of Science and Technology, N-7491 Trondheim, Norway; 5 Higher School of Economics, National Research University, 107078 Moscow, Russia

**Keywords:** aluminium, in utero exposure, birth outcomes, essential trace elements, neurotoxic elements, sex-dependent response to toxicants

## Abstract

Aluminium (Al) is a non-essential neurotoxicant and there is limited information regarding exposure to Al in utero. This study sought to evaluate the in utero exposure to Al in urban South African women, its effects on birth outcomes and possible synergistic effects between Al, essential and neurotoxic elements such as lead (Pb), mercury (Hg) and arsenic (As), as well as a a potential sex-dependent response to these elements in neonates. This study has found elevated levels of Al in urban women at delivery. The Spearman’s rank correlation coefficients (p-value) of the association between maternal serum Al and birth outcomes (gestational age and parity), and between maternal serum Al and Cu, Zn and Se, were statistically significant. However, in the general and the stratified models, no association was found between any of the birth outcomes and maternal serum Al. The association between maternal serum Al and neurotoxic elements at delivery showed a significant positive correlation for Pb only (rho = 0.361; *p* < 0.001) which was found to be sex-dependent in neonates (males, rho = 0.285; *p* < 0.004 and females, rho = 0.444, *p* < 0.001). Our preliminary findings indicate that in utero exposure to Al is an emerging concern requiring further research and directives from public health authorities.

## 1. Introduction

Aluminium (Al) together with lead (Pb), mercury (Hg) and arsenic (As) are recognised to be non-essential neurotoxicants with no known biological function in humans. They are predominantly environmental toxins that are known to affect brain cells or exhibit direct toxicity towards the genetic processes in neurons, such as transcription regulation and DNA repair mechanisms [1], although they have not been thoroughly studied or evaluated in humans. It has been reported that neurotoxicity resulting from the exposure to metals may alter DNA methylation, as already shown for methyl mercury (MeHg) [2,3]. Furthermore, studies investigating the possible synergistic effects of known neurotoxicants on human health are limited [1].

In the case of Pb, Hg and As, the toxicokinetics of these elements are well understood. However, in the case of Al, the mechanisms of absorption, metabolism, deposition and excretion are not yet fully defined since Al has been generally regarded as biologically inert. Research has shown that in humans Al can be absorbed and accumulated via dietary intake (including water and medications) and across the gastrointestinal tract [4] and by nasal inhalation which results in absorption across the olfactory epithelium [5]. On the other hand, the inhalation of particulate Al through the mouth may result in absorption across lung epithelia or the deposition of Al in the lung, followed by passage to the gut [6]. Dermal absorption of Al is yet another established route of exposure [7].

The distribution and accumulation of Al takes place in the whole blood, at both the organ and tissue level, as well as cellular level [8,9]. When Al intake exceeds the body’s excretory capacity, the excess is deposited in storage organs with the brain, bone, liver, heart and spleen being the primary targets. In addition, Al has the ability to cross the blood–brain barrier (BBB) as shown by various studies [10,11]. Exposure to high levels of Al is linked to a number of negative health impacts in humans such as neurological disorders, encephalopathy, bone disorders and microcytic anaemia [12]. Al is also thought to play a role in the aetiology of many diseases such as Alzheimer’s and Parkinsonism-Dementia [13,14,15]. In addition, a number of studies have shown that Al can affect the metabolism of essential metals in animals, occupationally exposed workers and pregnant women [16,17,18].

In modern times, Al is extensively used in engineering industries; as a flocculant in drinking water treatments; in food processing, packaging and cooking utensils; in cosmetics, pharmaceuticals and in hygiene products [16,19,20]. Our daily diet, medical and cosmetic preparations can contain significant amounts of Al that are absorbed through the gut [21]. It has been reported that the concomitant intake of acidic drinks such as fruit juices and cool drinks might add to absorption of Al [22]. In addition, the administration of citrate-containing calcium supplements to haemodialysis patients has been shown to increase Al absorption [23].

Thus, exposure to Al in the general population, including susceptible groups such as pregnant women, is increasing constantly but to date very few studies have quantified levels of exposure to Al in these populations. Most of the research on the reproductive health effects associated with exposure to Al are based on animal experiments but the findings from animal studies cannot be directly extrapolated to human exposures and outcomes [18,24,25,26,27].

The most recent research points to a possible role of Al in human reproduction with evidence of elevated concentrations of Al found in human semen, in spermatozoa, and in miscarried embryonic material [28,29].

Although very controversial, recent studies on the use and safety of paediatric Al adjuvant in vaccines to stimulate the immune response have come under scrutiny, mainly because of the acute exposure to Al and its potentially instant toxicity [30,31,32].

Even though it is well established that the unborn infant is at an increased risk of Al toxicity in utero due to its immaturity in terms of anatomical and physiological factors [33], to date prenatal exposure to Al has not been studied in any great depth in humans However, higher exposure to Al has been demonstrated in neonates and pre-term infants requiring parenteral nutrition or intravenous fluid therapy [34,35,36]. There is a real concern that Al exposure from parenteral nutrition in the high-risk pre-term infant may have adverse effects on bone health at a later stage, as well as on short-term cognitive outcomes [34]. These studies all reinforce the well-accepted vulnerability of the foetus, the neonate and the developing infant, to the toxic effects of Al exposure. Although it can be debated that the toxic effects of neurotoxic metals may result in responses that differ by sex, very few studies have investigated these responses in early life. For example, it has been reported that the male foetus is more susceptible to the neurotoxic effects of Pb and MeHg, while the female foetus is more susceptible to the immunotoxicity of Pb [37]. However, there is no indication in the literature of similar research being carried out for prenatal Al exposure.

Of greater concern is the perception that Al is a “safe” metal, and the lack of any legislation limiting human exposure to environmental Al, including women of reproductive age. Furthermore, the effects of concomitant exposures to Al and other neurotoxic elements have not been investigated in humans and early life.

The aim of the current study was to evaluate prenatal exposure to Al in urban coastal South African women; and to investigate the impact of prenatal Al exposure on birth outcomes and levels of selected trace elements. A potential synergistic effect was also investigated for exposures to Al and other exclusively neurotoxic elements (Pb, Hg, As). The possibility of a sex-dependent response to these concomitant exposures in neonates was also assessed.

This investigation is a part of the multidisciplinary, multi-institutional research collaboration between South Africa and Norway that evaluates prenatal exposures and effects of persistent toxic substances (PTS) on reproductive health. This project is carried out under the auspices of the Arctic Monitoring and Assessment Programme (AMAP).

## 2. Materials and Methods

### 2.1. Study Population

This cross-sectional study was performed in the urban centre of Cape Town, situated along the Atlantic coast of South Africa. Study participants were women who were admitted for delivery at the largest maternity public hospital in Cape Town. The procedure for inclusion in the study was as follows: at admission women were informed about the study by medical personnel on duty and a research assistant who distributed an information sheet/pamphlet about the study. Those who agreed to participate signed an informed consent form and agreed to donate blood before delivery, answer a socio-demographic questionnaire by interview and allow access to, and use of, data related to birth outcomes and birth complications, if any. The participation rate was high with more than 90% of women approached agreeing to participate in the study. Participation was voluntary and confidentiality was assured. Women were also informed that they could withdraw from the study at any time. A total of 200 women participated in the study.

### 2.2. Sample Collection and Analytical Procedures

From each woman 10 mL of venous blood was collected into a non-additive tube to obtain serum for analyses of Al and other trace metals. An additional 10 mL of maternal blood was collected into Ethylene diamine tetracetic acid (EDTA) tubes for the analyses of other metals (both trace and neurotoxic) using the Venoject sterile system and Becton, Dickinson & Company (BD, Franklin Lakes, NJ, USA) collection tubes. Blood for serum analyses was centrifuged and the serum was transferred into acid-washed polypropylene tubes with acid-washed plastic pipettes and frozen at −20 °C until analysed.

The Analyses of samples for the selected elements manganese (Mn), Hg, Pb and As in maternal whole blood have been described previously [17,38,39,40].

For the measurement of Al, Cu, Zn and Se in serum, samples were diluted 20-fold with a diluent (ammonia 2.5 mL; butanol 6 mL, 0.1% triton-X 50 µL and EDTA 50 µg in 500 mL deionized water). The following internal standards were also added to the diluent: indium (In, 25 µL), germanium (Ge, 25 µL), scandium (Sc, 25 µL), rhodium (Rh, 250 µL) and iridium (Ir, 250 µL). The instrument (Agilent inductively coupled plasma mass spectrometer (ICP-MS) 7900) was calibrated with calibration standards prepared in the diluent using a multi-element custom standard (SPECTRASCAN–SS028226). The concentrations of the standards for Al ranged from 0.1 to 50 µg/L, and for Cu, Zn and Se, from 0.1 to 100 µg/L. The internal standards used were Sc for Al and Ir for Se. Ge was used as an internal standard for both Cu and Zn.

All the samples were prepared in 15 mL Nunc^TM^ Trace Metal free tubes (Thermo Scientic, Johannesburg, South Africa). Blank samples were run in the beginning and after every 10 samples to check for carryover. The ICP-MS instrument (Agilent Technologies, Santa Clara, CA, USA) was run in general purpose mode, using helium gas. The mass/charge ratio of 27 was used for Al detection, and 63 and 66 was used for Cu and Zn, respectively. The integration time of 0.5 s was used for all three analytes. Each analyte was measure in triplicate, and reported as an average,. The average result was accepted if the RSD% was less than 10%.

The calibration curves were accepted if the r-squared value was greater than 0.999. A calibration verification standard (prepared from a different standard–LGC Custom multi standard-VHG-ZLGC1574-100) was run with every batch at 5 µg/L and 50 µg/L, with an acceptance criteria of 5%.

Two certified reference controls, Seronorm^TM^ Trace Elements Serum Level 1 (Lot no 11309438, Sero Ltd., Billingstad, Norway) and Level 2 (Lot no 1309416), were analysed with every analytical run in intervals of 10 samples for quality assurance of all element measurements. The limits of quantitation (LoQ) for Al, Cu, Zn and Se were 0.15, 0.06, 0.31 and 0.17 µg/L, respectively. Al was detected in all serum samples.

The % recovery of the certified controls was between 90 and 113 for both levels. The laboratory participates in the Royal College of Pathologists of Australasia (RCPA) quality-assurance programme for whole blood, serum and urine. The results obtained are consistently accepted with no indication of bias. All precautions to eliminate and prevent contamination at collection and during preparation of samples were applied throughout.

### 2.3. Covariates

Covariate information was obtained during the questionnaire-based interview and from medical records. Maternal weight, height, blood pressure and haemoglobin levels were recorded at the hospital on admission. From the medical records, the following neonate characteristics were retrieved: birth weight (g), birth length (cm), head circumference (cm) and gestational age (weeks), Apgar score at 1 and 5 min, and placenta weight (g). Pre-term labour was defined as mothers giving birth at less than 37 weeks of gestational age. Education was categorised as no education to completed primary school, completed secondary school and any level of tertiary education attained. Maternal tobacco smoking during pregnancy was defined as yes or no. Exposure to environmental tobacco smoke (ETS) was defined as exposure to tobacco smoke from smoking by others in the household. A binary classification was used for exposure to indoor smoke from the burning of fossil fuel (wood and coal) for the purpose of heating or cooking, separating study participants into those exposed to fossil fuel and those not exposed (for example, those using electricity). Dietary questions relating to the intake of proteins, carbohydrates, dairy products, tea, coffee, bottled water, vitamin supplementation, fruits, as well as vine, root and leafy vegetables, were assessed and classified as daily, at least once a week and seldom (both for pre-pregnancy and during pregnancy).

### 2.4. Statistical Analysis

The statistical analyses were performed using STATA (StataCorp, 2013. Stata Statistical Software: Release 13. College Station, TX, USA: StataCorp LP). Bivariate analyses between maternal serum Al exposure and covariates were evaluated by Spearman’s correlation coefficient. List-wise deletion was used so that the analysis was only run on cases which had a complete set of data. Most of the assumptions for linear regression were not met in this study’s data, therefore, non-parametric method quantile regression was used. Quantile regression analysis helps to understand the association between variables outside of the mean of the data unlike the ordinary least squares regression. It is carried out to understand outcomes that are non-normally distributed and that have non-linear relationships with independent variables [41]. Although, quantile regression can be used to test the group differences across the distribution (25th, 50th, 75th quantiles) of an outcome variable, this study focussed only on the 50th quantile i.e., the median of the outcome variable maternal serum Al [41]. All statistical tests were two-tailed and statistical significance was set at *p* < 0.05.

### 2.5. Ethical Considerations

The work was carried out in accordance with the Code of Ethics of the World Medical Association (Declaration of Helsinki). Ethics approval for the study was obtained from the Human Research Ethics Committee of the University of Witwatersrand in Johannesburg (Protocol no. M10742), and from the relevant provincial Department of Health. In addition, the chief executive officer (CEO) of the hospital had to confirm that he/she allowed the research work to proceed. Identical procedures were followed in terms of obtaining consent from participants. Confidentiality was maintained by assigning identification numbers to all study participants. During the informed consent process, it was emphasised that participation was voluntary and could be withdrawn at any time.

## 3. Results

### 3.1. Participant Characteristics

The background characteristics of the study participants are presented in Table 1. The majority of the mothers were single and of African Black ethnicity; 90% attained secondary and 7.5% tertiary education level. Most of the women resided in formal housing and owned the property, had access to electricity and to potable municipal tap water.

### 3.2. Obstetric and Birth Outcomes

Table 2 shows the descriptive data for obstetrics and birth outcomes. The mean age of mothers was 26 years and about 40% of women were primiparous. Haemoglobin levels, traceable in only 87 women, indicate moderate low concentrations. The average gestational age was 38.7 weeks, ranging from 24 to 44 weeks, with 58.1% of neonates being males.

### 3.3. Association between Exposure to Al, Maternal Covariates and Infant Anthropometry Measures at Birth

The preterm birth rate in this cohort was 14.88%. Although the data analyses in Table 3 showed no statistically significant association (in either direction) between preterm delivery and birth outcomes, this rate can be considered to be high, when compared with our previous studies in South Africa [15].

### 3.4. Concentration of Al, Selected Essential Elements and Neurotoxic Metals

Concentrations of Al, Cu, Zn and Se (in serum), and of Mn, Pb, Hg and As (in whole blood) of the study cohort at delivery are shown in Table 4. The average maternal serum concentration of Al (SD) was 25.5 (23.1) µg/L with geometric mean (GM) of 17.5 µg/L (95% confidence interval (CI): 14.8; 20.4). GM for Cu levels in serum was 2401 µg/L (95% CI: 2332; 2472) and for Zn levels in serum, 452.6 µg/L (95% CI: 449.05; 486.48). Se concentration in serum (GM) was 73.7 µg/L (95% CI: 70.9; 76.6) and GM of Mn in whole blood was 13.5 µg/L (95% CI: 12.8; 14.3).

The neurotoxic elements Pb, Hg and As were detected in all whole blood samples and concentrations were found to be considerably lower than those reported from other southern hemisphere regions [42].

### 3.5. Prenatal Sex-Specific Spearman’s Association between Maternal Serum Al and Essential and Neurotoxic Elements

For the total cohort, significant positive correlations were shown between maternal Al and Cu, Zn and Se (in serum), as well as between maternal Al in serum and Pb in whole blood, as seen in Table 5. After dividing the total cohort according to sex of neonates, a significant correlation was found between maternal Al serum and Cu (in serum) among male neonates, but none was found for female neonates. For both sexes, significant correlations were evident for Zn in serum (male, rho = 0.447; *p* < 0.001; female, rho = 0.402; *p* < 0.001). Concomitant exposure to Al in serum and Pb in whole blood was sex dependent for both sexes (male, rho = 0.285; *p* < 0.004; female, rho = 0.444; *p* < 0.001). No correlation was found between exposure to Al (in serum) and Hg or As (in whole blood).

### 3.6. Univariate and Multi-Variable Regression Analyses

Results of univariate and multi-variable quantile regression are shown in Table 6 (total sample), Table 7 (male neonates) and Table 8 (female neonates).

In the univariate regression, parity, eating of fruit during pregnancy, levels of Cu and Zn in maternal serum, and levels of Pb in the maternal blood were associated with high levels of maternal serum Al. However, after controlling for other factors (Table 6), only maternal serum Cu and Zn and maternal blood Pb were associated with increased maternal serum Al. After stratification of the model by sex of neonates, maternal serum Cu and Zn were positively associated with maternal serum Al, while placenta weight was negatively associated with maternal serum Al in the univariate analysis of male neonates (Table 7). After controlling for other factors, only maternal serum Zn was associated with maternal serum Al. In contrast, in the case of female neonates, maternal serum Zn and maternal blood Pb were positively associated with maternal serum Al, in both the univariate and multi-variable analyses (Table 8).

However, in the general and the stratified models, no association was found between any of the birth outcomes and maternal serum Al.

## 4. Discussion

The concentrations of Al in serum were found to be high when compared to published reference values for the general population [43]. According to the Agency for Toxic Substances and Disease Registry (ATSDR) of the US Department of Health and Human Services, Public Health Services, Atlanta, GA, USA, Al serum levels in healthy individuals who are not pregnant range from 1 to 3 μg/L [44]. The authors used the ATSDR reference values for the general population, as there is no equivalent for South African populations, mainly due to a very limited number of studies on aluminium exposure in this region. The mean Al (SD) level at delivery in this study was found to be 25.5 (23.1) μg/L, ranging from 0.18 to 60 μg/L and exceeding ASTDR normal levels. In addition, these Al concentrations in serum were 2.5 times higher (statistically significant) when compared to those of a cohort of South African rural women at delivery, in the previous study by the same investigators [17]. As anticipated, this urban coastal study population had a higher number of women who had secondary or tertiary education (97.5%), used electricity as fuel for cooking (97.5%) and had municipal water (67%) in their homes compared to the rural women (87.9%, 64.8% and 25%, respectively). It can be postulated that a number of factors contributed to the elevated exposure to Al in this urban coastal study cohort. It can be assumed that this urban coastal population had a higher standard of living, consumed an urban diet including ready-made meals, had an increased consumption of citrate-containing cool drinks, and frequently used cosmetics, various Al-containing hygiene products and Al-containing antacids and medications. Recently, it has been shown that prenatal vitamins and other supplements (both prescribed and self-medicated) sold in Canada and other countries contain various neurotoxic elements including Al, Pb, As, titanium, and thoreum [45]. The consumption of these products may pose a danger to the developing foetus, which has an immature detoxification mechanism when compared with its mother [45]. Thus, the mean concentration of Al in serum is higher in the urban coastal population, although the ranges are very similar in both populations, viz. 0.25 to 59.42 µg/L in rural women [17] versus 0.18 to 60 µg/L in urban coastal women (this study). Serum Al levels of over 100 µg/L are consistent with overload and reports show that levels above 100 µg/L indicate possible toxicity [46]. In the general and the stratified univariate and multi-variable quantile regression models, no association was found between any of the birth outcomes and maternal serum Al.

In a previous study of the rural cohort of women, no association was found for any of the anthropometry measures [17]. Likewise, a recent study in Jamaican neonates found no correlation between Al levels in cord blood and birth outcomes [47]. Based on the findings from the current study, we can suggest/postulate that exposure to high concentrations of Al during pregnancy may affect birth outcomes, although more studies are needed to confirm these preliminary associations between exposure to Al and birth outcomes.. The observed high preterm birth rate in the current study may be the result of behaviour/lifestyle factors. In South Africa, it is a common practice among some population groups to use traditional medication to induce labour prior to full-term pregnancy. In addition, smoking and drinking rates are higher in this cohort, when compared to our other study cohorts. It is well known that premature births worldwide have multi-factorial aetiologies, and arise because of complex pathways and influences. Examples of potential risk factors, which may or may not act synergistically, include infections, poor prenatal care, physical exertion, psychological stresses, multiple gestations, prior preterm birth, socioeconomic status, behavioural and lifestyle factors, race and ethnicity differences, etc.

The current study also measured the levels of selected essential trace elements at delivery. The concentrations of serum Cu, Zn, and whole blood Mn in urban coastal women (this study) were similar to those measured in rural South African women, in the previous study [17]. For both studies, the concentrations of Cu and Zn were above the reference values for the general population [48]. The levels of Se in serum and Mn in whole blood were similar to those reported in other studies (Mayo Medical Laboratories) [49]. Cu levels in serum are known to increase during pregnancy and double at full term. Other studies have shown significantly lower serum Cu levels in women with pathological pregnancies (e.g., habitual abortion, imminent abortion, missed abortion, missed labour, spontaneous abortion, and premature rupture of membranes) and postulated that lower levels of serum Cu in pregnancy could be predictors of some pathological pregnancies and should be included in routine prenatal diagnostics [50,51,52]. The reference range for serum Cu in pregnant females at delivery is 1180 to 3020 µg/L [46], compared to the range of our study participants of 1157 to 4281 µg/L. As our study population consisted mostly of participants of African ethnicity (60%), our findings are well aligned with those of a study on an African-American population whose Cu levels were found to be 8–12% higher than the reference range [46]. This does not necessarily mean that there is a biological reason for the similar Cu levels observed in the aforementioned study populations; it is merely interesting to note that both populations are from African ethnicity. The mean Cu serum concentration of 2451 µg/L in our study is quite similar to that found in a study on pregnant women in Turkey (2206.7 µg/L) [53] but the range in our study (1157 to 4281 µg/L) differs markedly from the range in a study in Spain (936 to 2304 µg/L) [54].

Pregnant women are at a higher risk of acquired Zn deficiency because of the high uptake of Zn by the foetus and associated tissues. However, the literature available on Zn levels during pregnancy is inconsistent as some reports indicate an increase in serum Zn levels, whereas other studies report no change, and some even reported a drop in Zn levels. Our study did not measure Zn levels before pregnancy, but the Zn levels at delivery of the majority of participants (98%) were found to be below the normal reference range of 700 to 1200 µg/L [46]. It has been reported that excessive use of prescribed iron and folic acid supplements during pregnancy interferes with Zn absorption and its utilization and may decrease the absorption of Zn [55,56]. In South Africa, pregnant women attending free or private prenatal care are prescribed these supplements; this was also the case with the population of this study. The mean value of 466.3 µg/L for Zn in our study is much lower than the mean values found in pregnant woman in Spain and China (758 µg/L; 710 µg/L), respectively [57,58]. In addition, two recent studies in China reported mean Zn concentrations of 5106 and 5434 µg/L; these levels are more than 10 times higher than the Zn levels found in our study [59,60].

Levels of Se were found to be very similar to those reported in other studies, 56 µg/L viz. 65 µg/L (Bermudez et al., 2015 and Liu et al., 2017), indicating sufficient intake of this element by our study population [57,58]. Maternal serum Al levels were positively associated with Cu, Zn, Se in serum; Cu, Se and Zn are important nutrients whose requirements increase with pregnancy. The intake of vitamins and essential mineral supplementation during pregnancy may also predispose the population to Al exposure which could possibly explain the positive correlation. In contrast, another study found that some vitamins, folic acid and iron supplements reduced biological concentrations of Al in pregnant women [61].

In utero exposure to neurotoxic elements is known to affect the development of the foetal central nervous system (CNS). In our study, we have investigated the possibility of a link between the concomitant exposure to Al and exclusively neurotoxic elements such as Pb, Hg and As. A significant positive correlation was found for Pb but not the other elements, indicating the potentiating toxic effect if pregnant women are exposed to both metals (Al and Pb). Remarkably, our study has also shown that concomitant exposure to Al and Pb was sex-dependent for male and female neonates. For female neonates, some negative associations were evident between Al levels in serum and Hg and As concentrations in whole blood, but these were not significant. In support of the findings of our study showing a sex-dependent association for Pb exposure in neonates, another study showed that foetal exposure to very low Pb levels may affect early cognitive domain, with boys being more susceptible than girls [62]. The latter study investigated the effect of a low-level Pb exposure, as measured between 20 to 24 weeks of pregnancy and in cord blood, on developmental scores up to 24 months of age in a group of children from the Polish Mother and Child Cohort. Since Al was not measured in their study, a direct comparison of their and our results is not possible; however, the results of both studies can serve as indicators of the effects of prenatal exposure to toxic metals. Interestingly, another study by the same investigators showed that prenatal Zn and Se status was associated with lower and higher child psychomotor abilities, respectively, within the first year of life [63].

The literature of the past few years indicates a susceptibility to toxicants and related biological factors that influence neurotoxicity, although much more research is necessary to further identify and understand the complex mechanisms contributing to different outcomes and effects from toxicant exposures in male and female neonates and infants. When investigating the neurotoxicity of several classes of chemicals (e.g., alcohols, heavy metals, pesticides, endocrine disruptors, and therapeutic agents), sex differences need to be considered to elucidate the possible genetic mechanisms that underlie individual differences in the effects of each toxicant [64]. Early-life exposure to Pb and MeHg is known to produce toxic effects in the foetus and studies have shown that males are more susceptible to neurotoxic effects, while females are more susceptible to the immunotoxicity of Pb [37]. Although both sexes are affected by Pb exposure, the incidence, manifestation, and severity of outcomes appear to differ. The sex-dependent variability in outcomes from Pb exposure during the developmental stages may arise from a combination of various influences such as intrinsic sex-specific molecular or genetic mechanisms and external risk factors like sex-specific responses to environmental stressors that may act through shared epigenetic pathways to influence the genome and behavioural output [65]. More research is needed; future studies should also include Al, given its involvement in neurotoxic and other health effects.

## 5. Conclusions

The current and previous study have not only measured Al exposure in pregnant South African women residing in coastal urban and coastal rural areas, but have also assessed the status of essential trace elements and its association with Al exposure. The findings from both studies have confirmed that exposure to Al at both low (rural women) and high (urban women) concentrations does affect the levels of essential trace elements. Exposure to high Al concentrations, as was the case in the current study on urban women, negatively affected two birth outcomes, namely head circumference and gestational age, with parity showing a positive association. These effects were not present in the cohort of rural women. Furthermore, the current study has shown a significant positive correlation between Al and Cu, Zn and Pb. For Pb, this correlation was also sex-dependent in both male and female neonates, supporting the evidence from the literature indicating sex-dependent variability in outcomes from Pb exposure during the developmental stages. In the multi-variable regression model, Pb remained positively associated with Al in female neonates, but no association was found in the male neonates. However, Zn remained positively associated with maternal serum Al in both male and female neonates. The shortcomings of the current study are the cross-sectional design and the low number of participants.

To date, prenatal and postnatal environmental exposure and toxicity of Al are not given due scientific recognition, and no attempts to create regulatory legislation have been forthcoming. Although very recent and quite controversial, an additional contributor to Al exposure being a health concern in neonates and infants is the fact that Al has become an adjuvant in some vaccines, to stimulate the immune response.

## Figures and Tables

**Table 1:** Socio-economic and dietary characteristics of participants.

Characteristic	Total (N = 200)
Age (Years) [mean, (standard deviation, SD)]	26.1 (6.3)
**Marital Status (%, n)**	
Married	37.1 (73)
Single/divorced/widowed	42.1 (83)
Co-habiting	20.8 (41)
**Education (%, n)**	
None/Primary	1.0 (2)
Secondary	90 (180)
Tertiary	7.5 (15)
**Race/Ethnicity (%, n)**	
African/Black	59.6 (115)
Others	40.4 (78)
**Percentage Unemployed (%, n)**	62.2 (122)
**Ownership of Home (%, n)**	
Owned	69.2 (135)
Rented	30.8 (60)
**Housing Type (%, n)**	
Formal housing	56.6 (111)
Flat	10.2 (20)
Backyard dwelling	9.2 (18)
Informal house (shack)	20.9 (41)
Others	3.1 (6)
**Source of Drinking Water**	
Municipal potable water—indoor tap	67.0 (132)
Municipal potable water—outdoor communal tap	33.0 (65)
Other (borehole and river)	0.0
**Fuel Used for Cooking (%, n)**	
Electricity	97.5 (192)
Paraffin	1.5 (3)
Gas/wood	1.0 (2)
**Fuel Used for Heating (%, n)**	
Electricity	41.5 (71)
Paraffin	29.8 (51)
Gas/wood/coal	4.1 (7)
None	24.6 (42)
**Prescribed Vitamin Supplements during Pregnancy**	93.2 (164)
**Ate Bread before Pregnancy (%, n)**	
Seldom/at least once a week	3.8 (7)
Almost everyday	96.2 (179)
**Ate Bread during Pregnancy (%, n)**	
Seldom/at least once a week	3.2 (6)
Almost everyday	96.8 (179)
**Root Vegetable before Pregnancy (%, n)**	
Seldom/at least once a week	10.0 (19)
Almost everyday	90.0 (172)
**Root Vegetable during Pregnancy (%, n)**	
Seldom/at least once a week	8.0 (16)
Almost everyday	92.0 (184)
**Ate Fruit before Pregnancy**	
Seldom/at least once a week	7.9 (15)
Almost everyday	92.2 (176)
**Ate Fruit during Pregnancy**	
Seldom/at least once a week	6.3 (9)
Almost everyday	93.7 (179)
**Bottled Water before Pregnancy**	
Seldom/at least once a week	56.5 (48)
Almost everyday	43.5 (37)
**Bottled Water during Pregnancy**	
Seldom/at least once a week	56.1 (46)
Almost everyday	43.9 (36)

**Table 2:** Obstetric and birth outcomes.

Characteristic	Total (N = 200)	Range
Maternal age (y) [mean, (SD)]	26.1 (6.3)	15–43
Maternal weight (kg) [mean, (SD)]	76.5 (19.7)	40–143
Maternal height (cm) [mean, (SD)]	156.9 (11.4)	138–183
Maternal haemoglobin (g/dL) [mean, (SD)]	11.04 (1.76)	6-15 (n = 87)
Maternal blood pressure at admission (mm Hg):		
BP systolic	122.6 (16.9)	100–160
BP diastolic	75.8 (12.3)	41–110
Gestational age (weeks) [mean, (SD)]	38.7 (2.9)	24–44
Birth weight (g) [mean, (SD)]	3080.9 (576.6)	855–4320
Birth length (cm) [mean, (SD)]	50.0 (4.3)	31–66
Head circumference (cm) [mean, (SD)]	34.6 (2.6)	27–50
Placenta weight (g)	617.4 (153.9)	320–1370
Apgar score 1 min [mean, (SD)]	8.1 (1.5)	2–10
Apgar score 5 min [mean, (SD)]	9.4 (0.9)	2–10
Sex (% male)	58.1	
Parity (%, n)		
0	40.2 (76)	
1+	59.8 (113)	

**Table 3:** Spearman’s rank correlation coefficient (*p*-value) of associations between maternal serum Al and birth outcomes (n = 135).

	Pre-Term (24–36 Weeks)		Term (37–44 Weeks)	
Birth Outcome	β	*p*-Value	β	*p*-Value
Birth weight	0.063	0.7912	−0.015	0.877
Birth length	0.143	0.547	0.039	0.682
Head circumference	0.154	0.517	−0.161	0.087
Gestational age	0.019	0.938	−0.188	0.044 *
Parity	0.156	0.512	0.194	0.038 *

* Statistically significant.

**Table 4:** Concentration of Al, selected essential and neurotoxic elements in whole blood or serum (as indicated).

Element	N	Mean (SD)	Range	GM	95% Conf. Interval	Median	95% Conf. Interval
Al serum (µg/L)	191	25.5 (23.1)	0.18–60	17.5	14.8;20.4	29.2	16.2;34.0
Selected essential elements							
Cu serum (µg/L)	192	2451 (51.1)	1157–4281	2401	2332;2472	2391.5	2315.7;2462.2
Zn serum (µg/L)	191	466.3 (120.6)	227.5–11295	452.6	449.05;486.48	445.3	435.3;473.0
Se serum (µg/L)	191	76.9 (28.7)	36.7–328.2	73.7	70.9;76.6	75.3	70.9;78.3
Mn blood (µg/L)	195	14.5 (4.9)	2.4–28.9	13.5	12.8;14.3	14.5	13.5;15.1
Selected neurotoxic elements							
Pb blood (µg/L)	195	12.7 (11.3)	5–62	9.5	8.6;10.5	10.0	5.0;11.0
Hg blood (µg/L)	191	1.5 (1.1)	0.3–6	1.1	0.9;1.2	1.2	1.1;1.5
As blood (µg/L)	195	0.5 (0.4)	0.1–3.3	0.4	0.3;0.4	0.4	0.4;0.5

N: total number of samples analysed per element differed.

**Table 5:** Spearman’s association between prenatal exposure to Al and sex-dependent response for essential and neurotoxic elements at delivery.

Elements	Total Cohort Rho (*p*-Value)	Male Rho (*p*-Value)	Female Rho (*p*-Value)
Selected essential elements			
Cu serum	0.285 (<0.001) *	0.360 (<0.001) *	0.152 (0.196)
Zn serum	0.435 (<0.001) *	0.447 (0.001) *	0.402 (<0.001) *
Se serum	0.195 (0.008) *	0.271 (0.006) *	0.070 (0.555)
Mn blood	0.043 (0.561)	0.023 (0.819)	0.098 (0.405)
Selected neurotoxic elements			
Pb blood	0.361 (<0.001) *	0.285 (0.004) *	0.444 (<0.001) *
Hg blood	−0.011 (0.881)	−0.001 (0.995)	−0.065 (0.586)
As blood	0.057 (0.444)	0.176 (0.075)	−0.118 (0.319)

* Statistically significant.

**Table 6:** Coefficient estimates of quantile regression at 50% quantile-univariate and multi-variable regression.

	Univariate			Multi-Variable		
Characteristic	Coefficient	*p*-Value	95% Conf.Int	Coefficient	*p*-Value	95% Conf.Int
**Head circumference**	–1.935	0.077	−4.078 to 0.208	-	-	-
**Apgar score 1 min**	4.173	0.022	0.604 to 7.741	-	-	-
**Apgar score 5 min**	4.282	0.163	−1.749 to 10.313	-	-	-
**Placenta weight**	0.006	0.752	−0.032 to 0.044	-	-	-
**Birth weight**	0.002	0.683	−0.007 to 0.011	-	-	-
**Birth length**	0.530	0.439	−0.820 to 1.880	-	-	-
**Parity**	5.708	0.005	1.770 to 9.645	-	-	-
**Ate fruit before pregnancy**						
Seldom/at least once a week	Reference					
Almost everyday	−12.600	0.068	−26.143 to 0.943			
**Ate fruit during pregnancy**						
Seldom/at least once a week	Reference					
Almost everyday	−18.450	0.022	−34.252 to 2.648			
**Bottled water before pregnancy**						
Seldom/at least once a week	Reference					
Almost everyday	12.775	0.106	−2.771 to 28.321			
**Bottled water during pregnancy**						
Seldom/at least once a week	Reference					
Almost everyday	15.560	0.060	−0.676 to 31.796			
**Root vegetable before pregnancy**						
Seldom/at least once a week	Reference					
Almost everyday	−10.310	0.297	−29.755 to 9.135			
**Root vegetable during pregnancy**						
Seldom/at least once a week	Reference					
Almost everyday	−14.130	0.187	−35.168 to 9.908			
**Ate bread before pregnancy**						
Seldom/at least once a week	Reference					
Almost everyday	9.770	0.553	−22.631 to 42.171			
**Ate bread during pregnancy**						
Seldom/at least once a week	Reference					
Almost everyday	11.305	0.530	−24.116 to 46.726			
**Cu serum**	0.014	0.009	0.004 to 0.025	0.008	0.044	0.0002 to 0.016
**Zn serum**	0.090	<0.001	0.058 to 0.123	0.070	<0.001	0.037 to 0.102
**Se serum**	0.149	0.120	−0.039 to 0.337			
**Mn blood**	0.598	0.307	−0.553 to 1.748			
**Pb blood**	0.680	0.002	0.258 to 1.103	0.437	0.014	0.090 to 0.783
**Hg blood**	3.503	0.180	−1.638 to 8.645			
**As blood**	6.967	0.281	−5.754 to 19.687			

**Table 7:** Coefficient estimates of quantile regression at 50% quantile-univariate and multi-variable regression for male neonates.

	Univariate			Multi-Variable		
Characteristic	Coefficient	*p*-Value	95% Conf.Int	Coefficient	*p*-Value	95% Conf.Int
**Head circumference**	−2.110	0.086	−4.531 to 0.306	-	-	-
**Apgar score 1 min**	4.540	0.032	0.392 to 8.688	-	-	-
**Apgar score 5 min**	6.880	0.058	−0.230 to 13.993	-	-	-
**Placenta weight**	−0.052	0.044	−0.103 to -0.001	-	-	-
**Birth weight**	−0.004	0.547	−0.018 to 0.010	-	-	-
**Birth length**	0.816	0.333	−0.850 to 2.481	-	-	-
**Parity**	5.409	0.044	0.159 to 10.658	-	-	-
**Ate fruit before pregnancy**						
Seldom/at least once a week	Reference					
Almost everyday	−7.940	0.442	−28.364 to 12.484			
**Ate fruit during pregnancy**						
Seldom/at least once a week	Reference					
Almost everyday	−17.085	0.295	−49.306 to 15.136			
**Bottled water before pregnancy**						
Seldom/at least once a week	Reference					
Almost everyday	15.370	0.099	−3.036 to 33.776			
**Bottled water during pregnancy**						
Seldom/at least once a week	Reference					
Almost everyday	7.780	0.048	0.088 to 15.472			
**Root vegetable before pregnancy**						
Seldom/at least once a week	Reference					
Almost everyday	−12.895	0.253	−35.129 to 9.339			
**Root vegetable during pregnancy**						
Seldom/at least once a week	Reference					
Almost everyday	−14.275	0.255	−39.026 to 10.476			
**Ate bread before pregnancy**						
Seldom/at least once a week	Reference					
Almost everyday			-			
**Ate bread during pregnancy**						
Seldom/at least once a week	Reference					
Almost everyday	11.184	0.610	−32.182 to 54.550			
**Cu serum**	0.022	0.001	0.009 to 0.034			
**Zn serum**	0.089	<0.001	0.048 to 0.129	0.089	<0.001	0.048 to 0.129
**Se serum**	0.141	0.185	−0.068 to 0.350			
**Mn blood**	0.531	0.436	−0.817 to 1.879			
**Pb blood**	0.460	0.108	−0.103 to 1.023			
**Hg blood**	3.429	0.270	−2.698 to 9.555			
**As blood**	11.42	0.102	−2.298 to 25.138			

**Table 8:** Coefficient estimates of quantile regression at 50% quantile-univariate and multi-variable regression for female neonates.

	Univariate			Multi-Variable		
Characteristic	Coefficient	*p*-Value	95% Conf.Int	Coefficient	*p*-Value	95% Conf.Int
**Head circumference**	−3.350	0.123	−7.627 to 0.927	-	-	-
**Apgar score 1 min**	0.990	0.713	−4.352 to 6.332	-	-	-
**Apgar score 5 min**	−1.451	0.697	−8.843 to 5.940	-	-	-
**Placenta weight**	0.033	0.218	−0.200 to 0.085	-	-	-
**Birth weight**	−0.004	0.573	−0.180 to 0.010	-	-	-
**Birth length**	0.626	0.549	−1.448 to 2.700	-	-	-
**Parity**	8.097	0.012	1.827 to 14.367	-	-	-
**Ate fruit before pregnancy**						
Seldom/at least once a week	Reference					
Almost everyday	−10.430	0.245	−28.158 to 7.298			
**Ate fruit during pregnancy**						
Seldom/at least once a week	Reference					
Almost everyday	−18.450	0.048	−36.725 to −0.175			
**Bottled water before pregnancy**						
Seldom/at least once a week	Reference					
Almost everyday	0.700	0.945	−19.810 to 21.210			
**Bottled water during pregnancy**						
Seldom/at least once a week	Reference					
Almost everyday	−0.670	0.907	−12.248 to 10.908			
**Root vegetable before pregnancy**						
Seldom/at least once a week	Reference					
Almost everyday	0.490	0.976	−32.397 to 31.417			
**Root vegetable during pregnancy**						
Seldom/at least once a week	Reference					
Almost everyday	−12.720	0.478	−48.235 to 22.795			
**Ate bread before pregnancy**						
Seldom/at least once a week	Reference					
Almost everyday			-			
**Ate bread during pregnancy**						
Seldom/at least once a week	Reference					
Almost everyday	16.800	0.525	−35.591 to 69.191			
**Cu serum**	0.011	0.173	−0.005 to 0.028			
**Zn serum**	0.092	0.002	0.0035 to 0.149	0.072	0.017	0.013 to 0.131
**Se serum**	0.118	0.389	−0.153 to 0.388			
**Mn blood**	0.807	0.374	−0.989 to 2.602			
**Pb blood**	0.970	0.005	0.310 to 1.631	0.632	0.048	0.004 to 1.259
**Hg blood**	1.406	0.679	−5.338 to 8.151			
**As blood**	−16.747	0.181	−41.452 to 7.957			
